# Comparison of two modes of vitamin B_12 _supplementation on neuroconduction and cognitive function among older people living in Santiago, Chile: a cluster randomized controlled trial. a study protocol [ISRCTN 02694183]

**DOI:** 10.1186/1475-2891-10-100

**Published:** 2011-09-27

**Authors:** Hugo Sánchez, Cecilia Albala, Lydia Lera, José Luis Castillo, Renato Verdugo, Manuel Lavados, Eva Hertrampf, Alex Brito, Lindsay Allen, Ricardo Uauy

**Affiliations:** 1 Public Health Nutrition Unit, Institute of Nutrition and Food Technology (INTA), University of Chile, El Libano 5524, Santiago, CP783490, Chile; 2 Department of Neurology, Faculty of Medicine, University of Chile, Av. José Miguel Infante N° 553, Santiago, CP7500691, Chile; 3 Department of Agriculture, Agricultural Research Services, Western Human Nutrition Research Center, University of California, Davis, CA, CP 95616, USA; 4 Department of Nutrition and Public Health Intervention Research, Faculty of Epidemiology and Population Health, London School of Hygiene & Tropical Medicine, Keppel Street, London WC1E 7HT, UK

**Keywords:** cobalamin, vitamin B12, cyanocobalamin, elderly, neurophysiology, cognitive disorders, nerve conduction, cluster randomized controlled trial, public health, Chile

## Abstract

**Background:**

Older people have a high risk of vitamin B_12 _deficiency; this can lead to varying degrees of cognitive and neurological impairment. CBL deficiency may present as macrocytic anemia, subacute combined degeneration of the spinal cord, or as neuropathy, but is often asymptomatic in older people. Less is known about subclinical vitamin B12 deficiency and concurrent neuroconduction and cognitive impairment. A Programme of Complementary Feeding for the Older Population (PACAM) in Chile delivers 2 complementary fortified foods that provide approximately 1.4 μg/day of vitamin B12 (2.4 μg/day elderly RDA). The aim of the present study is to assess whether supplementation with vitamin B12 will improve neuroconduction and cognitive function in older people who have biochemical evidence of vitamin B12 insufficiency in the absence of clinical deficiency.

**Methods:**

We designed a cluster double-blind placebo-controlled trial involving community dwelling people aged 70-79 living in Santiago, Chile. We randomized 15 clusters (health centers) involving 300 people (20 per cluster). Each cluster will be randomly assigned to one of three arms: a) a 1 mg vitamin B12 pill taken daily and a routine PACAM food; b) a placebo pill and the milk-PACAM food fortified to provide 1 mg of vitamin B12; c) the routine PACAM food and a placebo pill.

The study has been designed as an 18 month follow up period. The primary outcomes assessed at baseline, 4, 9 and 18 months will be: serum levels of vitamin B12, neuroconduction and cognitive function.

**Conclusions:**

In view of the high prevalence of vitamin B12 deficiency in later life, the present study has potential public health interest because since it will measure the impact of the existing program of complementary feeding as compared to two options that provide higher vitamin B12 intakes that might potentially may contribute in preserving neurophysiologic and cognitive function and thus improve quality of life for older people in Chile.

**Trial registration:**

ISRCTN: ISRCTN02694183

## Background and rationale

B12 is an essential micronutrient; in addition to its known effects on red cell maturation it plays multiple roles in metabolic pathways necessary for normal central and peripheral nervous system function. B12 plays a key role in one carbon metabolism (methyl group transfer) necessary for neurotransmitter, choline, nucleotides, and phospholipids synthesis [1]. Deficit is often seen in older people [2], in association with age-related gastric atrophy, low acid and intrinsic factor production resulting in malabsorption of this micronutrient [3-8].

Serum concentrations of vitamin B12 > 221 pmol/dL are considered normal, 148-221 pmol/dL corresponds to subclinical deficit, and levels < 148 pmol/dL as deficiency.

B12 deficiency presents clinical with abnormal neurologic signs, cognitive loss, peripheral neuropathy and psychiatric disorders. Sub-acute combined degeneration (SCD) of the spinal cord is the classical pathological finding of severe deficiency. Sensory polyneuropathy is also observed in B12 deficiency. This neuropathy typically involves preferentially large caliber afferent fibers [9]. If these manifestations are treated early, most symptoms disappear [10,11].

Clinical and electrophysiological features of symptomatic B12 deficiency with neurological manifestations have been reported [12,13]. However, subclinical B12 deficiency is less well characterized, with few studies and limited number of older people included [13,14]. This is relevant since population based surveys indicate that the prevalence of B12 deficiency in people > 60 years is 6-20% [15].

A need for routine vitamin B12 supplementation in older people in the absence of clinical deficit has not been established. The OPEN Study is currently evaluating the impact the B12 supplementation on neuroconduction and cognitive function in older people [16].

Chile has undergone a period of rapid demographic transition, which translates in an increase in the proportion of older people. Studies in free-living older people have been conducted in small groups of subjects; vitamin B12 deficit in these groups ranges from 7-51% depending on the definition of normal cut off values [17-19].

In an effort to promote healthy ageing, the Chilean Health Ministry initiated in 1998 the *Complementary Feeding Programme for the Older Population *(PACAM) providing a nutritional supplement, rich in micronutrients, to individuals ≥ 70 years registered at primary health centers. Since 2005, beneficiaries of PACAM receive B12 fortified foods providing approximately 1.7 μg/day, this is equivalent to 71% RDA [6].

Despite the high acceptability and adherence to PACAM by older people [20], vitamin B12 deficiency in beneficiaries reaches 35%, suggesting that the amount provided is low or that absorption is insufficient. There is agreement that the oral administration of B12 raises serum levels suggesting that there is a need to provide a higher dose. Thus the aim of this study is to determine whether the delivery of 1 mg vitamin B12 via PACAM has similar effectiveness to oral administration of 1 mg B12 as a pill; in terms of increasing serum levels, preserving cognitive function and neuroconduction in older people.

## Design and methodology

**Figure 1:**
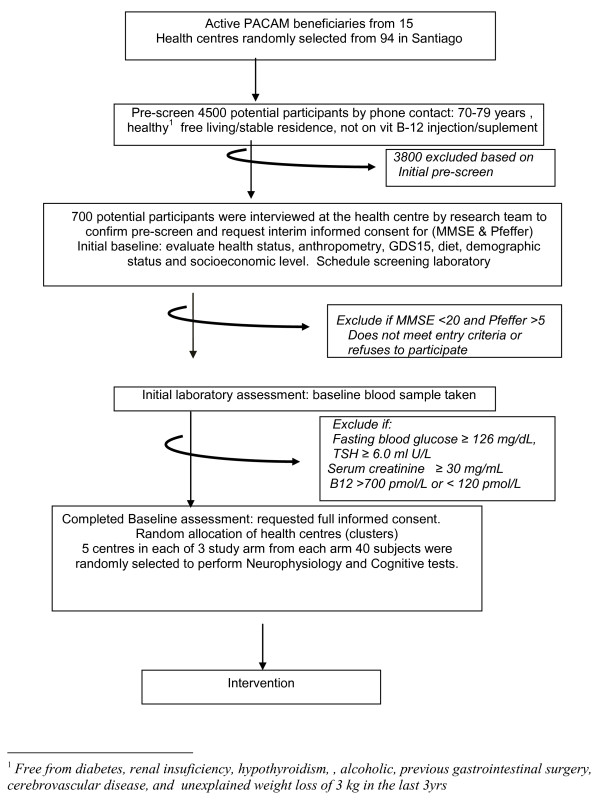
**Flow diagram for selection of participants, screening and baseline data collection**.

**Figure 2:**
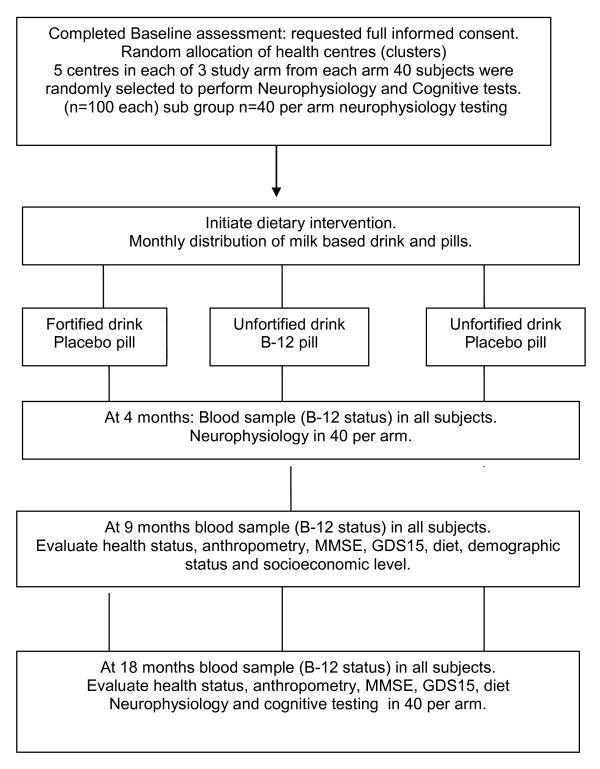
**Follow up scheme**.

The study is designed as a cluster double blind randomized placebo-controlled trial to evaluate two modes of high dose vitamin B12 delivery over an 18 months period in older people residing in Metropolitan Santiago, Chile. Figure 1 and 2 illustrate schematically the flow diagram for patient selection and procedures related to this research study, full description is presented as follows.

### Study hypotheses

The study aims to test the following hypotheses.

1. The vitamin B12 dose provided by PACAM to older people in Chile is insufficient to prevent vitamin B12 deficit and subclinical deficiency in the beneficiaries.

2. A dose (1 mg/day) of vitamin B12 intake provided in a food supplement or as a pill will correct subclinical deficiency vitamin B12 equally well, improving biomarkers of vitamin B12, cognitive function and neuroconduction in older people.

### Criteria for Cluster Inclusion and Selection

In Chile, elderly beneficiaries of PACAM receive special fortified foods through the National Health System primary health care centers. This trial was designed as a public health programme effectiveness study, thus the interventions were made available through the established health system under standard operating procedures, without the use of resource-intensive extraordinary efforts to secure uptake thus the design of the intervention considered the health center as the cluster unit for this study.

There are 94 health centers in Santiago Metropolitan Area. Considering that the target population is older people from low to medium socio-economic status, the nine health centers located in higher income municipalities were excluded. In order to facilitate data collection, health centers with less than 400 people aged 70.0-79.9 registered were excluded (n = 49) The geographic catchment areas of the remaining 36 health centers were mapped, and following further discussions and site inspections, 15 health centers were excluded because of insufficient interest among health center staff (self-perception of having heavy work burden), or poor infrastructure. 15 of 21 remaining health centers will be randomly selected and randomized, to one of the three treatment arms.

The project statistician will randomize clusters into the three study arms. Five centers will be included in each arm, 20 individuals in each center. Randomization will allow secure double blind allocation of center/cluster to one of the 3 arms of the study. Following random allocation a trial number will be given to each study participant for further analysis. A subsample of eight individuals will be randomly selected from each cluster for neurophysiological function tests

### Selection of participants from health centers

20 participants will be selected from each health center. Potential participants will be identified from the center registry.

### Participants Inclusion criteria

People aged 70 to 79 years, with documented residence and registration in the corresponding center where they collect the PACAM foods.

### Participant exclusion criteria

Phone calls will be used to identify and exclude those who are not free living or have self-reported medical conditions that increase susceptibility to vascular or neurologic damage such as diabetes, renal insuficiency, hypothyroidism, previous gastrointestinal surgery, cerebrovascular disease or any terminal condition.

Individuals with unexplained weight loss of 3 kg in the last 3 months and subjects who report use of vitamin B12 injection in the last year or current supplements will also be excluded.

### Recruitment of participants

Potentially eligible individuals, after securing they have no exclusion criteria by phone call; will receive an invitation explaining the nature and relevance of the study with a proposed appointment at the health center. The invitation will explicitly state that their refusal will not affect in any way their access to, or the quality of, health care they receive at the center. The research team will confirm the absence of exclusion criteria at the time of the health center interview, and will provide detailed information about the nature and relevance of the study and what will be involved if they agree to participate. Potential participants who manifest their interest will be asked to complete a survey on diet and mental health, and answer a short cognitive screen previously validated in Chile consisting of the Mini Mental State Examination (MMSE) plus the Pffefer Activities Questionnaire (PFAQ) [21]. Potential participants will have a MMSE score > 19 (maximum of 30) plus a PFAQ score less than six. Potential participants will be asked to give informed consent and provide a blood sample and, if eligible based on the results will be contacted. Those unwilling to provide consent will be thanked for their time and cooperation. Potential participants providing full informed consent will be asked to provide a 13 ml venous blood sample to assess serum vitamin B12, hemoglobin, mean red cell volume (MCV), glucose, TSH and serum creatinine concentrations. Blood samples will be sent to a central laboratory for analysis. Serum will be kept to assay B12 status and serum folate in the definitive participants. Eligible participants are defined as those with fasting blood glucose < 126 mg/dL, TSH < 6.0 mlU/L, creatinine clearance < 30 ml/min (calculated using Cockcroft equation) [7], and serum vitamin B12 120-700 pmol/L.

The research team will provide the results of the blood tests to all potential participants. All eligible participants will be contacted by phone by the study coordinator who will restate the nature and relevance of the study and what will be involved if they agree to take part. They will be told that a prerequisite of their joining the study will be that they agree to consume PACAM foods and a daily tablet, and not receive any vitamin B12 injection over the course of the study. They will be informed that non-involvement will not prejudice the health care provision from their general practice. At this point, potential participants will be visited at home to give them the opportunity to ask any questions before being invited to give full informed consent to take part in the study.

A subsample of 120 subjects (8 per cluster, 40 per arm) will undergo neurophysiological tests. The baseline tests will be performed at the Department of Neurological Sciences, Faculty of Medicine University of Chile. The full battery will take approximately 75 minutes to complete. The study manager will arrange private transportation for the participant to attend the appointment. The use of private transportation is expected to have a positive impact on attendance and compliance rates. Individuals unwilling to participate at the baseline hospital appointment, will be thanked for their time and co-operation.

### Intervention

A routine fortified food program provided monthly, delivered by the Chilean Ministry of Health under PACAM through the health centers. The nutritional products are 1 kg of "*Años Dorados" *(a cereal-legume and vegetable powdered food) which provides 400 kcal/100 g and multiple micronutrients (including 0,5 μg/100 g vitamin B12) and 1 kg of "*Bebida Láctea"*, a micronutrient fortified milk-based drink (including 2.8 μg/100 g vitamin B12) monthly. The recommended serving size of these supplements provides 1.4 μg daily corresponding to 58% of the daily vitamin B12 recommended intake and 20% of daily energy needed by older people [17]. Following the completion of baseline testing, participants will be introduced to the intervention. All subjects in the three study arms will receive both a pill and the food supplement in order to maintain the double blind nature of the study. Each center/cluster was randomly assigned to one of three arms: one of the arms will receive a 1 mg vitamin B12 pill taken daily and a routine PACAM food, a second arm will be provided with a placebo pill and the "*Bebida Lactea*" (milk-PACAM food fortified) to provide 1 mg of vitamin B12 as consumed on a daily basis; finally a third arm will receive the routine PACAM food and a placebo. The pill will be identical in size, shape, color, smell and taste for both the intervention and the placebo arms of the trial. The intervention pill and the fortified milk base drink will each contain 1 mg of B12.

We choose to fortify the milk delivered by the program, given the higher adherence to "*Bebida Lactea*" and "*Años Dorados*" by older people and the low intra-family dilution of the products [22].

Adherence to the nutrition intervention is defined for this study as collecting 1 kg per month of the fortified food as documented by the health centers. Adherence for the pill intervention will be monitored based on collection of the pills supplement from the health center, 12 out of 18 months will be considered acceptable adherence Vitamin B12 content of fortified and control *Bebida Lactea *will be monitored in a sample randomly obtained at every health center.

### Outcome measures

#### Primary outcomes

##### Serum vitamin B12 status

Serum vitamin B12 will be assessed in duplicate at baseline, 4, 9 and 18 months by the SimulTRAC_SNB radioassay kit (^57^Co/Folate^125^I) (MP Diagnostics, Orangeburg, NY).

##### Peripheral nerve conduction

Velocity conduction of the right and left fibular nerve will be measured using standard techniques at baseline, 4 and 18 months.

##### Minimental score

Mini Mental State Examination (MMSE) is an easy to administer test, and has been validated for Chilean elderly. It will be performed at baseline and at 18 months.

#### Secondary outcomes

##### Vitamin B12 status and serum folate

Total homocysteine in plasma (tHcy), serum methyl malonic acid (MMA), holo- transcobalamin (Holo-TC) and serum folate will be assayed at baseline, 4, 9 and 18 months. MMA will be analyzed by LC/MS/MS using a modification of the method of Kushnir et al (18). Specifically, the plasma aliquot is reduced from 1 mL to 25 mL and methyl tert-butyl-ether (MTBE) is reduced from 3 mL to 0.4 mL, allowing the use of disposable 1.5 mL microcentrifuge tubes and eliminating the need for centrifugation. MMA calibration standards (25-1000 nM) and d3-MMA surrogate (500 nM) are prepared in DI water and frozen until use. Incubation is increased from 5 min at 50° to 30 min at 60° to maximize derivatization efficiency. The chromatographic interference between MMA and succinic acid (SA) is removed (eliminating the need to correct MMA for SA interference) by changing the mobile phase from 85% methanol and 15% 5 mM ammonium formate aqueous buffer to 53% methanol and 47% 1.67 mM ammonium formate, increasing the column temperature from 40° to 60°, reducing flow rate from 0.75 mL/min to 0.25 mL/min, and increasing the time between injections from 1 min to 2 min. tHcy will be determined by HPLC with a fluorescence detector (Agilent 1200, Santa Clara, CA) and serum Hl-TC with the Axis Shield HoloTC RIA (Axis-Shield Diagnostics Ltd., Dundee, UK). Analysis of the blood samples will be the responsibility Department of Agriculture, Agricultural Research Services, Western Human Nutrition Research Center, Davis, CA, USA. Blood samples will be couriered on dry ice in batches from INTA, Santiago-Chile to Davis, California. Haematological and biochemical parameters will be assessed at Micronutrients Laboratory, INTA, University of Chile.

##### Peripheral nerve conduction

Nerve conduction studies of the right and left sural, right and left tibial, right median and fibular nerves will be performed using standard techniques at baseline, 4 and 18 months. Nerves will be stimulated supramaximally at proximal and distal sites and conduction velocity in the distal leg nerve segments calculated. Supramaximal nerve stimuli are routinely used daily to investigate neurological conditions with minimal discomfort.

##### Neurological battery

Evaluation includes sensory pathways of conduction of afferent sensory pathways of small diameter, quantitative thermal and somatosensory responses, specific neurosensory pathways and visual evoked potential. All techniques will use surface electrodes and limb temperature will be controlled to be above 30°C by suitable heating and blankets and will be applied at baseline, 4 and 18 months.

##### Visual evoked potentials (VEPs)

VEPs (Nicolet Vicking Quest) will be obtained using a checkerboard pattern subtending an angle of 59 minutes of arc with a stimulation rate of 2 Hz. The active electrode was at Oz with a Cz reference and a ground at Fpz. Two trials of 200 responses were recorded for each eye. An upper limit of 120 msec will be considered as normal for the P100 potential (mean 2.5 SD).

##### Somatosensory evoked potentials (SSEPs)

SSEPs will be performed bilaterally in the median and tibial nerves. Stimulus duration will be 0.1 msec. Median nerve SSEPs will be recorded over the scalp (N20), in C3'/C4'-Fpz, on the cervical spine (N13) and at the Erb point (N9). Central conduction time (CCT) will be calculated between the cervical spine and the contra lateral cortex (N13-N20). In our laboratory the upper normal limit for CCT is 6.8 msec. Tibial nerve SSEPs (N/P37) will be recorded at Cz'-Fpz and in the popliteal region. Normality will be defined by a cortical response with an absolute latency within 2.5 standard errors from height-latency regression line. Sensory nerve conduction of both sural nerves will be performed following conventional techniques.

##### Cognitive function

Language ability will be assessed with the Boston Naming Test, which has a range from 0 to 60. Verbal memory will be evaluated from recall of ten words from the battery developed by the Consortium to Establish a Registry for Alzheimer's disease (CERAD), testing delayed recall and recognition. The score ranges from 0 to 10 and is repeated three times in each subject, for a possible maximum of 30 points. The Letters and Numbers test from the WAIS-II-R evaluated working memory and the Digit Symbol subtest (DSST) of the Wechsler Intelligence Scale for Adults scored attention. Executive function will be measured with the Stroop Test. The Geriatric Depression Scale (GDS-15) evaluated symptoms of depression, defined as > 6 points. They will be performed at baseline and 18 months.

### Sample size

Based on the capacity to detect changes in serum vitamin B12, MMSE and fibular nerve conduction velocity as the main outcomes, a sample size of 300 individuals in total (100 per arm) was calculated. In the case of serum vitamin B12 (criteria for measuring efficacy at 4 mo and effectiveness at 18 mo), the sample size was calculated using Chi square statistical test to compare proportions. We considered a frequency of vitamin B12 deficiency (vitamin B12 < 221 pmol/L, IOM/USA criteria) of 41%, in accordance with previous studies performed by our group [23]. Considering that we will give a supplement of 1 mg of vitamin B12 per day, we expect a reduction of approximately 50% in the prevalence of vitamin B12 deficiency in the intervention group. A sample size of 84 subjects is required to detect a drop from 41% to 20,5% in vitamin B12 deficiency in the intervention group, with a significance of 95% (α: 0.05) and a power of 80% (β = 0.20),. Considering a possible loss of 15% at 18 months, the number of subjects in each arm is 97. Using an intra-cluster coefficient (ICC) of 0.0001 [24], and an estimated number of 20 older adults per cluster, it will be necessary to select 5 clusters per arm, corresponding to a total of 300 older adults.

The sample size for neuroconduction was based on changes in velocity nerve conduction of fibular nerve (criteria for measuring efficacy at 4 months and effectiveness at 18 months). The calculation was performed using two tailed student t test. We used a mean velocity of 44.3 ms and a SD of 4.37 in accordance with previous studies performed by our group. In order to detect an 8% change in the velocity of conduction in the intervention group (considered clinically relevant), with a significance of 95% (α: 0.05) and a power of 90% (β = 0.10) a sample size of 26 subjects is required. Considering a possible loss of 15% at 18 months, the number of subjects in each group is 30. Using an ICC of 0.01499 obtained in a previous study and an estimated number of 8 older adults per cluster, 4 clusters per arm are necessary.

The sample size for the outcome MMSE scores (criteria for measuring effectiveness) is based on a two-tailed student t test. To detect an expected loss of 1.5 points in MMSE score at 18 months with a significance of 95% (α: 0.05) and a power of 80% (β = 0.20) a sample size of 44 subjects is required. The calculation was performed using the average MMSE score of 27.0 and a SD of 2.5 in accordance with previous studies performed by our group. Considering a possible loss of 15% at 18 months, the number of subjects per each group was 51 older adults. Using an ICC of 0.04171, obtained in a previous study and an estimated number of 20 older adults per cluster, 5 clusters per arm are necessary.

In summary, for the outcomes serum vitamin B12 and MMSE 100 older people (5 clusters 20 people each) per arm will be recruited. For the nerve conduction outcome 40 older people (5 clusters 8 people each) per arm will be recruited.

### Trial monitoring

The main aim of monitoring is to ensure that participants adhere to the study, and secondly to minimize attrition. A field worker will call the participants by telephone every 2 weeks during the first 4 months of follow up (8 calls in total), this will serve to remind participants of the importance of study and the need to comply with the regular consumption of products.

### Assessment at baseline, 4, 9 and 18 months

All participants will be assessed for:

1. CBL status and serum folate: as at baseline, 4 m, 9 m, 18 m

2. Nerve conduction: as at baseline, 4 m, 18 m

3. Clinical markers of neurological function: as at baseline, 4 m and 18 m

4. Cognitive health: as at baseline, 18 m

5. Anthropometry: as at baseline, 9 m, 18 m.

### Data analysis

Statisticians at INTA will conduct data analysis; all involved in this analysis will be blind to the treatment allocation. At the end of the interventions, primary analyses will be carried out based on the groups as randomized ("intention to treat"). Results will be presented as appropriate effect sizes with a measure of precision (95% confidence intervals). All analyses will take account of the clustered design. Covariates will be adjusted for in the analysis as necessary. Further exploratory analyses will be based on those participants who fully follow the various treatment protocols ("per-protocol analyses").

### Ethical approval

The Institutional Review Board at INTA, University of Chile, approved the protocol for this study.

### Trial organization

### Investigators

1. Hugo Sánchez: epidemiologist based at INTA, University of Chile.

2. Cecilia Albala: senior geriatric epidemiologist based at INTA, University of Chile.

3. Eva Hertrampf: senior haematologist based at INTA, University of Chile.

4. José Luis Castillo: clinical neurologist based at Department of Neurology, Faculty of Medicine, University of Chile.

5. Renato Verdugo: clinical neurologist based at Department of Neurology, Faculty of Medicine, University of Chile.

6. Manuel Lavados: clinical neurologist based at Department of Neurology, Faculty of Medicine, University of Chile.

7. Lydia Lera: statistician based at INTA, University of Chile.

8. Alex Brito: nutritionist based at INTA, University of Chile.

9. Lindsay Allen: senior public health nutritionist based at Department of Agriculture, Agricultural Research Services, Western Human Nutrition Research Center, Davis, CA, USA.

10. Ricardo Uauy (Principal Investigator PI): senior public health nutritionist based at INTA, University of Chile and Department of Nutrition and Public Health Intervention Research, Faculty of Epidemiology and Population Health, London School of Hygiene & Tropical Medicine, London, UK.

#### Project Management Group

A Project Management Group (comprising principal investigators, trial manager, research nurses, database manager and statistician) will run the trial on a day-to-day basis to ensure the smooth operation of the project. Regular review meetings will be held with other members of the team as appropriate.

#### Publication Policy

The protocol and the primary results of the trial will be published with authorship in relation to specific participation in the study, with name order to be presented by the PI. Suggested revisions in order of authors will meet approval of the PI. Co-investigators in their area of expertise subject and the PI will lead publications in specific areas of the study or on methodological aspects. The requirements for authorship will follow recommended practice in journal guidelines.

#### Confidentiality

All the research team, including practice staff, will be made aware of the need for full confidentiality when dealing with patient information; participants will be identified by their study number to ensure confidentiality. However, as the participants in the study will be followed up for 18 months following randomization, it is essential that the teams at INTA and the Department of Neurology, University of Chile have a record of their names and addresses in addition to the allocated trial number. Stringent precautions will be taken to ensure confidentiality of participant's names and addresses. The investigators and local coordinators will ensure that records are kept confidential and access is restricted.

#### Sponsor

The Institute of Nutrition and Food Technology and the Department of Neurology at University of Chile will act as the main sponsors for this study. Delegated responsibilities will be assigned locally.

## Conclusion

Vitamin B12 deficiency is common in older people, it can often be asymptomatic or present with clinical signs of deficiency. The current trial is designed to test the hypothesis that subclinical deficiency vitamin B12 is associated with impaired cognition and neuroconduction in older people. This study will reveal whether a daily high-dose of vitamin B12 will correct and prevent deficiency, will modify biomarkers of vitamin B12 status and will attenuate cognitive decline and impaired neuroconduction in older people.

## Abbreviations

CMAP: Compound muscle action potential; CERAD: Consortium to Establish a Registry for Alzheimer's Disease; FONDECYT: Chilean National Science and Technology Research Fund; Holo-TC: Holo-transcobalamin; ICC: Intra-cluster coefficient; MCV: Mean Cell Volume; MTBE: Methyl tert-butyl-ether; MMSE: Mini Mental State Examination; PI: Principal Investigator; PMG: Project Management Group; PACAM: Programme of Complementary Feeding for the Older Population; RDA: Recommended dietary allowances; USDA: United States Department of Agriculture; SAP: Sensory action potential; MMA: Serum methyl malonic acid; SSEPs: Somatosensory evoked potentials; TSH: Thyroid stimulating hormone; tHcy: Total Homocysteine; VEPs: Visual evoked potentials.

## Competing interests

The authors declare that they have no competing interests.

## Authors' contributions

RU conceived the study. HS, CA, EH, ML, RV and RU were applicants for the funding. All authors contributed to designing the study and drafting the protocol. All authors read and approved the final protocol.
